# Improved contraceptive use among women and men in Uganda between 1995-2016: A repeated cross-sectional population study

**DOI:** 10.1371/journal.pone.0219963

**Published:** 2019-07-18

**Authors:** Amrita Namasivayam, Sarah Lovell, Sarah Namutamba, Philip J. Schluter

**Affiliations:** 1 School of Health Sciences, University of Canterbury—Te Whare Wānanga o Waitaha, Christchurch, New Zealand; 2 Institute of Public Health, Makerere University, Kampala, Uganda; 3 School of Clinical Medicine—Primary Care Clinical Unit, The University of Queensland, Brisbane, Australia; FHI360, UNITED STATES

## Abstract

**Background:**

Research on contraceptive behaviour changes over time in Uganda is scarce, yet it has among the highest fertility and maternal mortality rates of any country in the East African region. Understanding temporal patterns of contraceptive use for both women and men is vital in evaluating the effectiveness of family planning interventions and strategies, and identifying those with the most unmet need. Using repeated nationally representative cross-sectional samples, this study charts the changes in Uganda’s population-based contraceptive use over recent years.

**Methods:**

Five Demographic and Health Survey datasets for Uganda over 21 years, from 1995 to 2016, were sourced and interrogated. Eligible participants included all women aged 15–49 years and men aged 15–54 years. Responses to questions on modern and any (modern or traditional) contraceptive use were analysed. Stratified by gender, weighted regression analyses were employed to detect change over time. The patterns associated with key demographic variables were also investigated.

**Results:**

Overall, 50,027 women and 14,092 men were included within the study. In 2016, 30.3% of women and 39.9% of men were using any contraceptive method, a significant non-linear increase from 13.4% of women and 20.3% of men in 1995. Furthermore, 27.3% of women and 35.9% of men were using modern contraceptive methods in 2016, an increase from 7.4% of women and 10.4% of men in 1995. All considered demographic variables were significantly associated with contraceptive use for both women and men (all *P<*0.001); and for women, all variables differentially changed over time (all *P<*0.001).

**Conclusion:**

This study showed a significant increase and dynamism across key demographic variables in contraceptive uptake by both women and men. Sustained family planning programs and interventions have successfully resulted in behaviour change across the Ugandan population. However, continued efforts are needed to further reduce Uganda’s relatively high fertility and associated maternal mortality rates.

## Introduction

Contraception, or family planning, allows women, men and couples to choose if and when to have children by way of voluntarily and intentionally delaying, spacing or limiting pregnancies [1]. Thus, contraception has been, and continues to be, a key focus of the global agenda for maternal health. Access to and use of contraception by women and men can improve the health, economic, and social domains of their lives [2]. Spacing or limiting pregnancies allows for improved health outcomes for a mother and her child, together with better financial and resource management resulting from smaller, healthier families, and reduced child care demands [3]. Delayed childbearing increases a woman’s likelihood of higher educational attainment, and better employment prospects, a higher level of financial independence and empowerment, as well as reducing the risks and complications associated with early pregnancies [2, 3]. Contraceptive use also prevents or reduces the likelihood of high-risk and unintended pregnancies, which can lead to unsafe abortions and adverse maternal health outcomes. Despite the successes and advances thus far, estimates for the number of women in developing regions who have an unmet need for contraception (those who are sexually active and want to avoid, space or limit pregnancies, but who are not using modern contraception methods) stood at 214 million in 2017 [2].

Uganda has one of the highest fertility and maternal mortality rates in the East African region, estimated at 5.4 births per woman in 2016 [4] and 343 maternal deaths per 100,000 live births in 2015 [5], respectively. Taken together, these figures underscore the high maternal health burden faced by Ugandan women of reproductive age [2]. Uganda also consistently has one of the lowest contraceptive use prevalence rates among East African countries [6]. It was estimated that only 39% of married women of reproductive age used contraception in 2016 [7]. Consequently, Ugandan women frequently report having more children than they desired, and short intervals between pregnancies [8]. This places both the health of the mother and her newborn child at risk, as well as economically impacting larger families by straining financial resources, and reducing access to education and healthcare [3].

Although Uganda has seen many directed initiatives and improvements in the distribution provision, access and uptake of contraceptive services over the last decade, particularly in community-based distribution [9] and the use of injectable methods [10], unmet need remains unacceptably high [8]. Currently, a combination of public, private and non-governmental organizations (NGOs), sometimes referred to as implementing partners (IPs), deliver family planning services to urban and rural populations [11]. Different IPs operate and partner in various capacities based on region and role; some engage in direct service provision and/or provide mobile outreach services, while others partner with local clinics to supply and fund contraceptive services. These services are usually provided free of charge, or at a nominal cost in private clinics, but travel and waiting times may be long [12], and availability limited [13]. Other IPs provide training in family planning counselling and service provision of short-term methods (such as: condoms, pills, and injectables) for village health teams and community health workers, who serve as the first tier of healthcare access for many communities [14].

Previous studies in Uganda have been predominantly qualitative in nature, focusing on factors influencing contraceptive use, and barriers contributing to unmet need among women; see, for example [12, 15, 16]. Few, if any, quantitative studies have considered men and their contraceptive use in Uganda, despite the acknowledged need for such information [17]. In the context of reproductive health and contraception, men need to be considered as they are not only women's partners, but individuals with distinct reproductive histories and desires of their own. In many societies, men are key decision-makers but often not directly involved in contraceptive discussions or programs [18]. Men’s attitudes and behaviours around contraceptive use thus directly impact fertility and maternal mortality rates. Tracking the temporal patterns of contraceptive use for both women and men is critical to understand the success of initiatives aimed at improving their use and to inform future health promotion policies in Uganda. To date, such epidemiological studies have not been forthcoming.

This study analyses changes in contraceptive use among women and men of reproductive age in Uganda, using five representative Uganda Demographic and Health Surveys (DHS) datasets. The primary aim is to describe patterns in any and modern contraceptive use among women aged 15–49 years and men aged 15–54 years for 21 years, from 1995 to 2016. The secondary aim is to investigate these patterns over a set of purposefully selected key demographic factors, namely: age, education, place of residence, and region.

## Materials and methods

### Study design and setting

A repeated, nationally representative, cross-sectional population study of women and men of reproductive age in Uganda, using a stratified (urban/rural) two-stage cluster design.

### Participants

All women aged 15–49 years who were either permanent residents of the selected households or visitors who stayed in the household the night before the survey were eligible to be interviewed. In one-third of the sampled households, all men aged 15–54 years, including both usual residents and visitors who stayed in the household the night before the interview, were also eligible for individual interviews. Data for women and men were collected across all surveys using the standardized DHS women’s and men’s questionnaires, respectively.

### Primary variables

The DHS definition of unmet need for contraception was employed, referring to women who “(i) are not pregnant and not postpartum amenorrhoeic and are considered fecund and want to postpone their next birth for two or more years or stop childbearing altogether but are not using a contraceptive method, or (ii) have a mistimed or unwanted current pregnancy, or (iii) are postpartum amenorrhoeic and their last birth in the last two years was mistimed or unwanted” [4]. The two principle outcome variables in this study were ‘*Any contraceptive use*’ and ‘*Modern contraceptive use*’. These were elicited and presented separately for women and men. Based on responses to the question “Are you or your partner currently doing something or using any method to delay or avoid getting pregnant?”, and “If yes, which method are you using?”, the DHS variable ‘contraceptive use and intention’ had already been created within all of the DHS datasets. The variable ‘Any contraceptive use’ was then created based on responses to this DHS variable ‘contraceptive use and intention’; responses were dichotomized into Yes (uses a modern or traditional method), and No (not using a method but intends to use later, does not intend to use, never had sex). For ‘modern contraceptive use’, responses for the same variable were dichotomized into Yes (uses a modern method), and No (uses a traditional method, not using a method but intends to use later, does not intend to use, never had sex). Modern methods referred to any of the following: female sterilization, male sterilization, oral contraceptive pills, the intrauterine contraceptive device (IUD), injectables, implants, male and/or female condoms, diaphragms, contraceptive foam and contraceptive jelly, and lactational amenorrhea method (LAM), and other modern contraceptive methods (including cervical cap, contraceptive sponge, and others). Traditional methods referred to periodic abstinence (rhythm/ calendar method), or withdrawal. Any contraceptive use refers to any specific method (modern or traditional), including female sterilization. Women’s and men’s responses to any or modern contraceptive use included methods used by their partner, as well as methods requiring couple negotiation (such as condom use or abstinence).

### Selected key demographic variables

Age grouping, education level, place of residence and region of residence were all purposefully selected as being key factors associated with contraceptive use, and important in charting its change over time. Here, age was grouped as 15–19, 20–24, 25–34, 35–39, and ≥40 years; highest educational attainment (attendance) was classified into no education, primary, secondary or higher; place of residence was dichotomized into urban and rural; while region of residence was defined by North, West, East, and Central geographic locations.

### Procedure

This study utilized data from the DHS, which are nationally representative population-based surveys. These repeated, country-wide cross-sectional surveys are commissioned by the United States Agency for International Development (USAID) and periodically carried out by the governments of different countries, with operational support from ICF International. Datasets are available through application to MEASURE DHS (https://dhsprogram.com/data/).

The DHS employ a stratified (urban/rural) two-stage cluster design. The first stage involves enumeration areas (EAs) initially being randomly selected from the most recent population census sample frame; each EA covers a geographic area with an average of 130 households. The second stage involves the systematic selection of households from the EAs. The surveys use standardized questionnaires developed by the MEASURE DHS program specifically for women, men and households; these are administered during face-to-face interviews. Detailed information about sampling methodologies and data collection procedures can be found from the MEASURE DHS webpage (https://dhsprogram.com/data/), or DHS reports for respective countries [19].

### Statistical methods

Analyses were undertaken separately on the women’s and men’s datasets. Reporting of analyses were informed by the STROBE guidelines (www.strobe-statement.org) [20]. Unweighted frequencies were reported, together with associated weighted percentages. Weighting accounted for the stratified two-stage cluster design to provide population estimates. A time variable was calculated for the years since 1995 (the baseline year). For the primary objective, first (time) and second (time^2^) order weighted quadratic regressions were employed to model the rate of change in contraceptive use over the study period. The statistical superiority of the quadratic regression model over its linear counterpart was assessed via the log-likelihood test, using unweighted data. For the secondary objective, weighted logistic regression models were employed for each key demographic variable. For each variable, the main effect and time interaction terms were introduced together within the model, along with the time and time^2^ variables. Suppose the independent variable of interest is labelled “*X*” and the contraceptive use outcome of interest is labelled “*Y*”, this implies that the logistic regression takes the form:

$$logit(Y)=intercept+\beta_{1}\times time+\beta_{2}\times {time}^{2}+\beta_{3}\times X+\beta_{4}\times X\times time$$

so that *β*_3_ gives the estimate of the main effect and *β*_4_ gives the time interaction term. Measures of association were presented as odds ratios (ORs) and 95% confidence intervals (CIs). Analyses and graphing were undertaken using specialist statistical software Stata SE version 15.1 (StataCorp, College Station, TX, USA), and significance was defined by α = 0.05.

### Ethical considerations

Data collection questionnaires and processes for the DHS surveys are reviewed and approved by the ICF Institutional Review Board (IRB). Additionally, country-specific DHS survey protocols are reviewed by the ICF IRB and by an IRB in the host country. As a part of the DHS survey methodology and ethics process, informed consent is obtained from all participants prior to their participation in the survey, and the collection of information is done confidentially. The datasets used did not carry any personal identifiable information and permission to use them were obtained from MEASURE DHS. Once a data request has been approved, no further ethical clearance is required from the ICF IRB. The study complied with the ethical standards for human experimentation as established by the Helsinki Declaration and New Zealand’s Health and Disability Ethics Committee (HDEC). HDEC defined this study as minimal risk observational research and it did not require ethics committee review. All methods and reporting were performed in accordance with HDEC’s relevant guidelines and regulations.

## Results

### Participants

Individual data were elicited from 7,070 women in 1995, 7,246 in 2000/2001, 8,531 in 2006, 8,674 in 2011, and 18,506 women in 2016, totalling 50,027 women overall. Similarly, individual data were collected from 1,996 men in 1995, 1,962 in 2000/2001, 2,503 in 2006, 2,295 in 2011, and 5,336 men in 2016, totalling 14,092 men altogether. Response rates varied across the years from 93.8% (2011) to 97.0% (2016) for women, and from 85.1% (2000/2001) to 90.7% (2006) for men. For all participants across all years, the response rates were higher in rural areas. The main reason cited by the DHS final survey reports for non-response was the failure to find the eligible participants in their homes, despite repeated visits to the household.

### Demographic characteristics

The characteristics of women and men between 1995 and 2016 for the selected key demographic variables in this study appears in Tables A-D in S1 File. Among women across all years of the study (Tables A and B in S1 File), the largest proportion of respondents were aged 15–19 years, had attained primary education, and resided in rural areas.

Among men across all years of the study (Tables C and D in S1 File), ages were more evenly spread over the different groups compared to the corresponding distribution for women. Similar to women, the majority of men had attained at least primary education; although compared to women, a larger proportion of men attained a secondary education or higher.

### Contraceptive use over time

**Fig 1:**
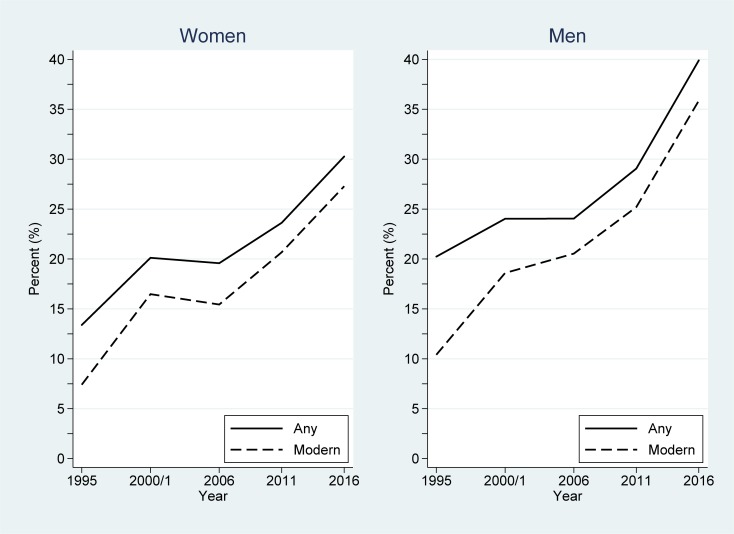
Changes in any and modern contraceptive use among women and men in Uganda across the study period (1995–2016).

In 2016, 30.3% of women, and 39.9% of men of reproductive age in Uganda were using any contraceptive methods, an increase from 13.4% of women and 20.3% of men in 1995. Furthermore, 27.3% of women and 35.9% of men were using a modern method of contraception in 2016, an increase from 7.4% and 10.4%, respectively, in 1995. Fig 1 depicts contraceptive use among women and men in Uganda across the study period. Non-linear increases in any and modern contraceptive use patterns are suggested in Fig 1, both for women and men.

Weighted quadratic regression analyses confirmed these non-linear increases in any and modern contraceptive use over time. Table 1 gives the estimated coefficients and associated 95% CIs derived from these analyses, together with the level of significance for each term. For any contraceptive use, the quadratic model was significantly better than its linear counterpart for both women (*P<*0.001) and men (*P*<0.001), demonstrating rapid, non-linear increases over the study period. For women, the predicted rate of any contraceptive use was given by 0.147 + 0.0035×(year–1995) + 1.7E-4×(year–1995)^2^. For men, the predicted rate of any contraceptive use was given by 0.213 –- 0.0031×(year–1995) + 5.6E-4×(year–1995)^2^. Similarly, for modern contraceptive use, the quadratic model was significantly better than its linear counterpart for both women (*P*<0.001) and men (*P*<0.001), also demonstrating rapid, non-linear increases over the study period. Here, the predicted rate for women was 0.090 + 0.0062×(year–1995) + 1.1E-4×(year–1995)^2^, whereas for men the predicted rate was given by 0.116 + 0.0053×(year–1995) + 2.9E-4×(year–1995)^2^.

**Table 1:** Estimates together with associated 95% CIs for regression models of any and modern contraceptive use among women and men in Uganda over the years 1995 to 2016.

		Base year	Years since 1995			
		(1995)^†^	Linear term		Quadratic term	
Contraceptive use		est.	est.	(95% CI)	est.	(95% CI)
*Women*						
	Any	0.147	0.0035	(0.0007, 0.0062)*	1.7E-4	(5.9E-5, 3.0E-4)**
	Modern	0.090	0.0062	(0.0038, 0.0086)***	1.1E-4	(2.6E-7, 2.2E-4)*
*Men*						
	Any	0.213	-0.0031	(-0.0076, 0.0014)	5.6E-4	(3.6E-4, 7.6E-4)***
	Modern	0.116	0.0053	(0.0014, 0.0092)**	2.9E-4	(1.0E-4, 4.7E-4)**

Note:

^†^first year of the study period; E denotes times 10 to the power of (e.g.: 1.7E-4 is 1.7×10^−4^ which is 0.00017); level of significance denoted by

*P<0.05

**P<0.01

***P<0.001.

### Women’s contraceptive use over time by key demographic variables

Among women using either any method, or specifically a modern method of contraception, the largest proportion of users were between the ages of 30–34 and 35–39 years (for the years 1995-2000/2001, and 2011), and 25–29 and 30–34 years (for the years 2006 and 2016) (Tables A and B in S1 File); those with a secondary education or higher; those living in an urban area; and women living in the Central region of the country. Conversely, across all the study years, women aged 15–19 years had the lowest proportion of contraceptive use, as did women with no education and those living in a rural area.

**Fig 2:**
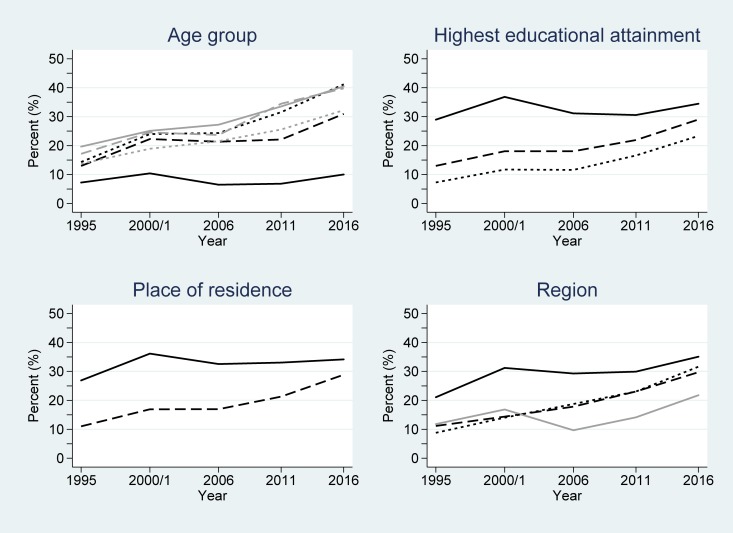
Changes in any contraceptive use among women in Uganda across the study period (1995–2016), partitioned by the considered demographic variables. Key: age group (years): 15–19 (black/solid); 20–24 (black/dash); 25–29 (black/dot); 30–34 (grey/solid); 35–39 (grey/dash); ≥40 (grey/dot); highest educational attainment: secondary and higher (solid); primary (dash); none (dot); place of residence: urban (solid); rural (dash); and region: Central (black/solid); East (dash); West (dot); North (grey/solid).

**Fig 3:**
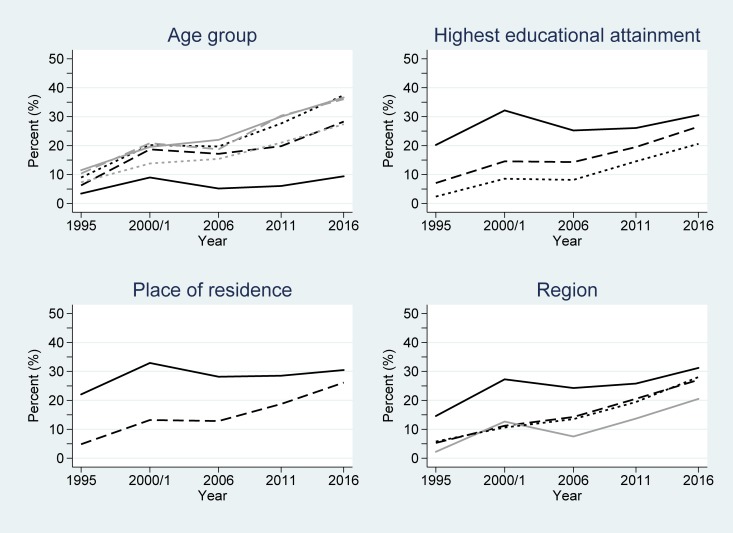
Changes in modern contraceptive use among women in Uganda across the study period (1995–2016). Key: age group (years): 15–19 (black/solid); 20–24 (black/dash); 25–29 (black/dot); 30–34 (grey/solid); 35–39 (grey/dash); ≥40 (grey/dot); highest educational attainment: secondary and higher (solid); primary (dash); none (dot); place of residence: urban (solid); rural (dash); and region: Central (black/solid); East (dash); West (dot); North (grey/solid).

Each of the key demographic variables were separately related to any and modern contraceptive use by women in logistic regression models. All variables, and their interactions over time, were significantly associated with contraceptive use after adjusting for the time and time^2^ changes (all *P*<0.001) (Table 2). This implies that the significant difference in the likelihood of contraceptive use within each demographic variable observed at baseline (1995) also significantly changed over time (Figs 2 and 3). Considering any contraceptive use by educational attainment groupings, women with a secondary or higher educational attainment had odds 5.84 greater of using contraception than those without any education at baseline (1995); see Table 2. However, this gap narrows over time–seen by the interaction term <1.0 (in Table 2) and the lines converging in Fig 2. By 2016, the estimated odds of any contraceptive use for women with secondary or higher educational attainment was exp(ln(5.84) + ln(0.94)×(2016–1995)) = 1.59 greater than that of women without any education, after accounting for the general non-linear increase in any contraceptive use over time. This implies that over the 21 year period, women without any education have had a relatively faster contraceptive use uptake than more educated women (although their actual usage still lagged behind).

**Table 2:** OR together with associated 95% CIs of using any and modern contraceptive method for women and men in Uganda over the years 1995–2016.

		Women				Men			
		Main effect		Time interaction		Main effect		Time interaction	
Contraceptive use		OR	(95% CI)	OR	(95% CI)	OR	(95% CI)	OR	(95% CI)
**Any contraception**									
*Age (years)*			*P*<0.001		*P*<0.001		*P*<0.001		*P* = 0.42
	15–19	1	(reference)			1	(reference)		
	20–24	2.16	(1.75, 2.66)	1.03	(1.02, 1.05)	3.51	(2.60, 4.72)	0.99	(0.98, 1.02)
	25–29	2.27	(1.84, 2.81)	1.05	(1.04, 1.07)	2.99	(2.15, 4.16)	1.00	(0.98, 1.02)
	30–34	2.98	(2.43, 3.65)	1.04	(1.02, 1.05)	3.66	(2.47, 5.41)	0.99	(0.96, 1.01)
	35–39	2.60	(2.07, 3.26)	1.04	(1.03, 1.06)	3.34	(2.39, 4.68)	0.99	(0.96, 1.01)
	≥40	2.04	(1.65, 2.51)	1.04	(1.03, 1.05)	2.73	(1.96, 3.81)	1.00	(0.98, 1.02)
*Highest educational attainment*			*P*<0.001		*P*<0.001		*P*<0.001		*P* = 0.41
	None	1	(reference)			1	(reference)		
	Primary	1.90	(1.59, 2.27)	0.98	(0.97, 1.00)	1.97	(1.29, 3.00)	1.00	(0.97, 1.02)
	Secondary or higher	5.84	(4.77, 7.14)	0.94	(0.93, 0.96)	4.63	(3.02, 7.11)	0.99	(0.96, 1.02)
*Place of residence*			*P*<0.001		*P*<0.001		*P*<0.001		*P*<0.001
	Urban	1	(reference)			1	(reference)		
	Rural	0.29	(0.25, 0.34)	1.05	(1.04, 1.06)	0.31	(0.25, 0.38)	1.03	(1.02, 1.05)
*Region*			*P*<0.001		*P*<0.001		*P*<0.001		*P* = 0.004
	Central	1	(reference)			1	(reference)		
	East	0.38	(0.30, 0.47)	1.04	(1.02, 1.05)	0.58	(0.43, 0.77)	1.01	(0.99, 1.02)
	North	0.37	(0.29, 0.47)	1.01	(1.00, 1.03)	0.55	(0.40, 0.76)	1.01	(0.99, 1.03)
	West	0.32	(0.25, 0.41)	1.05	(1.03, 1.06)	0.37	(0.27, 0.50)	1.03	(1.01, 1.05)
**Modern contraception**									
*Age (years)*			*P*<0.001		*P*<0.001		*P*<0.001		*P* = 0.76
	15–19	1	(reference)			1	(reference)		
	20–24	2.16	(0.75, 2.66)	1.03	(1.02, 1.04)	2.97	(2.11, 4.17)	1.00	(0.98, 1.02)
	25–29	2.51	(2.00, 3.13)	1.04	(1.03, 1.06)	2.21	(1.54, 3.18)	1.01	(0.99, 1.04)
	30–34	2.94	(2.35, 3.68)	1.03	(1.02, 1.05)	2.17	(1.48, 3.17)	1.00	(0.98, 1.02)
	35–39	2.77	(2.15, 3.57)	1.04	(1.02, 1.05)	2.09	(1.43, 3.06)	1.00	(0.98, 1.03)
	≥40	1.86	(1.44, 2.40)	1.04	(1.02, 1.05)	1.80	(1.25, 2.58)	1.01	(0.99, 1.03)
*Highest educational attainment*			*P*<0.001		*P*<0.001		*P*<0.001		*P*<0.001
	None	1	(reference)			1	(reference)		
	Primary	2.50	(2.03, 3.10)	0.97	(0.96, 0.98)	2.40	(1.36, 4.25)	0.99	(0.95, 1.02)
	Secondary or higher	8.66	(6.87, 10.92)	0.92	(0.91, 0.94)	8.22	(4.62, 14.62)	0.96	(0.92, 0.99)
*Place of residence*			*P*<0.001		*P*<0.001		*P*<0.001		*P*<0.001
	Urban	1	(reference)			1	(reference)		
	Rural	0.20	(0.16, 0.24)	1.07	(0.06, 1.08)	0.19	(0.15, 0.25)	1.06	(1.04, 1.08)
*Region*			*P*<0.001		*P*<0.001		*P*<0.001		*P*<0.001
	Central	1	(reference)			1	(reference)		
	East	0.30	(0.23, 0.38)	1.05	(1.04, 1.07)	0.39	(0.28, 0.54)	1.03	(1.00, 1.05)
	North	0.19	(0.14, 0.26)	1.05	(1.03, 1.07)	0.14	(0.09, 0.22)	1.08	(1.05, 1.11)
	West	0.28	(0.21, 0.36)	1.06	(1.04, 1.07)	0.23	(0.16, 0.34)	1.05	(1.03, 1.08)

### Men’s contraceptive use over time by key demographic variables

Among male users of any method of contraception, the largest proportion of users in 1995 were between the ages of 30–34 and 35–39 years. This gradually changed over time to men aged 20–24 and 25–29 years being the largest user groups in 2011 and 2016 (Table C in S1 File). As with women, men with a secondary education or higher, living in an urban area, and within the Central region of the country comprised the largest proportion of users of any contraceptive method. For modern contraceptive use across the study period, men aged 20–24 years remained the largest user group by age. Similar to the use of any contraceptive method, men with a secondary education or higher, living in an urban area and within the Central region of the country were the largest user groups of modern contraceptives.

**Fig 4:**
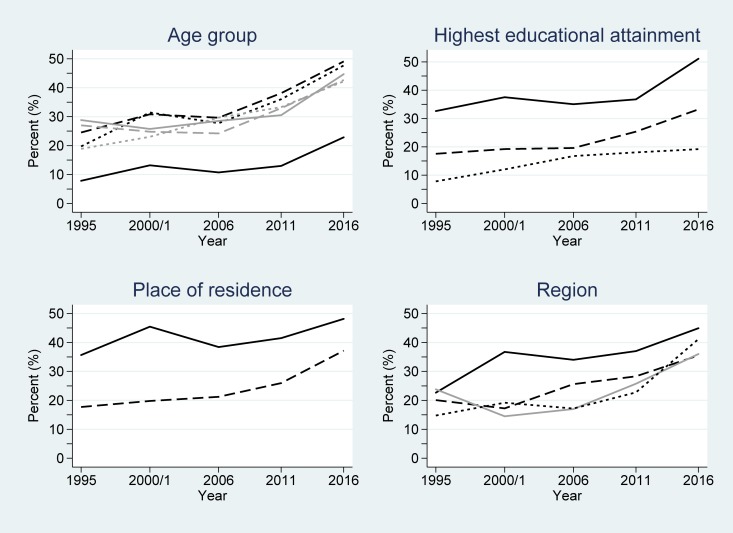
Changes in any contraceptive use among men in Uganda across the study period (1995–2016), partitioned by the considered demographic variables. Key: age group (years): 15–19 (black/solid); 20–24 (black/dash); 25–29 (black/dot); 30–34 (grey/solid); 35–39 (grey/dash); ≥40 (grey/dot); highest educational attainment: secondary and higher (solid); primary (dash); none (dot); place of residence: urban (solid); rural (dash); and region: Central (black/solid); East (dash); West (dot); North (grey/solid).

**Fig 5:**
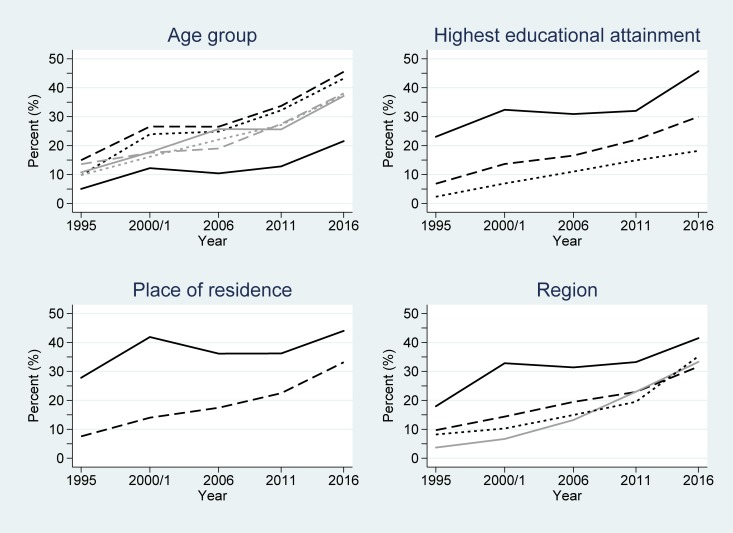
Changes in modern contraceptive use among men in Uganda across the study period (1995–2016). Key: age group (years): 15–19 (black/solid); 20–24 (black/dash); 25–29 (black/dot); 30–34 (grey/solid); 35–39 (grey/dash); ≥40 (grey/dot); highest educational attainment: secondary and higher (solid); primary (dash); none (dot); place of residence: urban (solid); rural (dash); and region: Central (black/solid); East (dash); West (dot); North (grey/solid).

Table 2 also shows the logistic regression model results for men. Similar to women, the main effect terms were significant for all the considered variables in any contraceptive and modern contraceptive use models (all *P*<0.001). However, unlike women, there was no significant interaction over time for any contraceptive use among men grouped by age (*P* = 0.42) or highest educational attainment (*P* = 0.41) or for modern contraceptive use by age (*P* = 0.76). This implies that the significant differences observed at baseline were largely preserved over time for these variables (Figs 4 and 5). Considering any contraceptive use by educational attainment groupings, men with secondary or higher educational attainment had odds 4.63 greater of using any contraception than those without any education at baseline (1995) (Table 2). By 2016, the estimated odds of any contraceptive use for men with secondary or higher educational attainment was exp(ln(4.63) + ln(0.99)×(2016–1995)) = 3.75 greater than that for men without any education, after accounting for the general non-linear increase in any contraceptive use over time. Although less, this estimated OR was non-significantly smaller–and there was little convergence seen between the associated lines in Fig 4.

## Discussion

In this first epidemiological study of its kind, it was demonstrated that both any and modern contraceptive use among women and men has significantly increased over time in Uganda and, particularly noteworthy, that the rate of change of contraceptive use has accelerated in latter years compared to earlier years. This non-linear increase in latter years may be partially explained by the end of the war between the Ugandan government and the Lords’ Resistance Army in 2006, and the resumption and restoration of the country’s healthcare system and services [21].

The more recent and rapid increase in both women and men’s contraceptive use has important implications. Though men’s reported contraceptive use also includes methods used by their partners, the increase in the percentage of men using condoms, together with a corresponding decrease in the percentage of men’s contraceptive non-use over the study period, is significant. This is particularly so, given that condom use has been largely associated with HIV prevention and protection efforts in the past [22], and is still more commonly linked with casual sexual relationships rather than monogamous partnerships [23]. However, as previous research on male attitudes toward contraception have shown, communication and educational campaigns via mass media and community-led initiatives can significantly increase contraceptive use, including condom use [24]. Given the increased outreach and programmatic efforts around family planning in Uganda in recent years, these increases seen among men likely reflect a shift in attitudes and receptivity towards contraceptive use. It is also important to acknowledge the continued and widespread covert contraceptive use among women in Uganda; in many instances, men may not even be aware of their partners’ use of contraceptives [25]. Therefore, the figures for men’s contraceptive use in this study speak directly to their own awareness and choice to use contraception within their sexual relationships.

Although men’s participation in the family planning process has been recognized as being critical to its effectiveness, traditional gender norms and perceptions often dictate that pregnancy, family planning and reproductive health are a woman’s ‘domain’ or ‘business’, and thereby exclude men’s involvement in the process [15, 18, 25]. Yet, partner opposition is often a significant predictor of poor healthcare access, unmet need for contraception, the use of traditional rather than modern methods, and clandestine use of contraception [25–28]. The largely patriarchal nature of Ugandan society and gender norms around male-dominant, normative decision-making processes, as well as the lack of spousal communication on fertility preference, and the timing and spacing of pregnancies [18, 27, 29, 30] often results in men making decisions about contraception without much discussion or consultation with their partners [26, 31–33]. Contraceptive use has been shown to be higher in communities where women have more autonomy and decision-making power [34], and where spousal communication takes place, whether direct or indirect [26].

The structural limitations of some family planning programs further exacerbate the gendered differences health service use; for example, providers being female, men not feeling welcome or comfortable, and a lack of trust for providers and not being assured of their confidentiality during discussions about family planning, often result in men not participating at all [25, 35]. Similarly, campaigns around family planning often target women [15]. Given that relatively few studies have previously looked at contraceptive use among men in Uganda, and the focus of these studies have largely been on barriers to male involvement, the results presented here are very encouraging, indicating the success of, and support for current efforts to improve male involvement in family planning and reproductive health.

The changes in contraceptive use associated with the different demographic variables for women and men across the study period also point to important differential shifts in behaviour within the population at large. The changes observed among the groupings by educational level and place of residence are of particular note. The relatively steeper increase in contraceptive uptake seen among women and men with no education, along with those with only a primary education, compared to those with a secondary education or higher may reflect the success of these family planning programs in effectively reaching lower educated populations [36]. However, it must also be noted that these population’s baseline contraceptive usage rates were substantially less than their more educated counterparts and still remained so by the study’s end date. In terms of place of residence, given that the majority of Uganda’s population is rural and at times very hard to reach [37], accessibility to reproductive health services is often challenging [12, 13]. Community health workers and village health teams are often the critical first touchpoint in these settings, providing services such as family planning counselling and at times, short-term family planning methods [38]. These increases in contraceptive may reflect the success of family planning initiatives and outreach programs effectively reaching these rural and remote communities.

The temporary decrease in contraceptive use seen in the data for both women and men in 2006 is likely to be a result of the end of the long-standing war in Uganda in the same year, and the subsequent suspension and withdrawal of humanitarian aid from the country. A secondary contributing factor to the observed decrease in contraceptive use in 2006 could also have been the shift in focus of HIV prevention campaigns, from the Abstinence-Be Faithful-Condom use (ABC) approach, to predominantly abstinence [22]. In the early and mid-2000s, many HIV/AIDs campaign efforts and prevention programs also withdrew from Uganda, due to the country’s rapid HIV prevalence decline in the early 1990s [39]. The intensity of the promotion and distribution of condoms, which was a large component of these campaigns, therefore likely decreased or slowed temporarily, before the country’s public healthcare system resumed these efforts as a continuing aspect of reproductive health programs.

### Strengths and limitations

The utilization of large, nationally representative datasets lends robustness to the results, as does the repeated, cross-sectional study design, using variables that were consistently measured using the same questionnaires and sampling techniques across the study period. Another strength of this study is the investigation of contraceptive use among men, a largely understudied population. Women and men are both important players in understanding and improving contraceptive use by, and for, each other. The study is also novel in following the changes in contraceptive use among women and men for purposefully selected variables. This allows the impact of initiatives on those most at need to be assessed, and inform health policy decision-makers about groups at high risk of not using contraception.

While the study has these salient strengths, it also has weaknesses. Data collected for all the variables were self-reported, hence subject to recall bias and response bias, and the psychometric properties of the tools were not readily available. While the DHS response rates were relatively good, ranging from 85.1% to 97.0%, those who did not participate are likely to have lower contraceptive use and poorer health-seeking behaviours than those who did participate. This may over-inflate the usage estimates reported within. Moreover, the geographical regions were consistent across the study years, except in 1995, where the Kitgum district was not included; in 2000/2001, where the districts of Kasese and Bundibugyo in the Western Region and Gulu and Kitgum in the Northern Region were excluded (due to access issues around security at the time); and in 2006, where parts of the northern region were oversampled in order to provide estimates for two areas of interest: Karamoja and internally displaced persons camps. In 2016, three additional land areas in greater Kampala, as well as islands and mountain regions were included. Given that the combined population of these districts corresponded to <5% of the total Ugandan population at each of the respective time points, the sampling bias associated with the inclusion/exclusion of these districts is likely to be negligible. Finally, it must also be emphasised that the results presented in this paper can only be applied to the study period between 1995 and 2016. Contraceptive usage rates should not be extrapolated to years outside this study period as it is unlikely that the observed non-linear patterns would continue beyond this timeframe.

## Conclusions

The findings from this study point to the success of family planning programs and interventions in Uganda that have resulted in important population behaviour change. This empirical evidence also serves to inform future reproductive health programs and policies, aligning with the Uganda Family Planning Costed Implementation Plan 2015–2020. This study shows a significant increase and dynamism across key demographic variables in contraceptive use and uptake by both women and men. Consequently, to better understand the predictors of current contraceptive use in Uganda, as well as their associated effect sizes and impact, updated epidemiological modelling is necessary–as past models are no longer likely to provide a valid representation. The findings contained herein, together with any further such epidemiological investigations, are vital in evaluating the past and informing future evidence-based strategies and directions of family planning programs and initiatives. Ultimately, it is hoped that these results will aid in reducing Ugandan women’s unacceptably high maternal health burden.

## Supporting information

S1 File Tables A-D.
(DOCX)
