# Supplementary material for: Improved contraceptive use among women and men in Uganda between 1995-2016: A repeated cross-sectional population study
Source: PLoS One. 2019 Jul 18;14(7):e0219963. doi: 10.1371/journal.pone.0219963 (PMC6638941; doi:10.1371/journal.pone.0219963)
Supplement: S1 File — Tables A-D. (DOCX) [file pone.0219963.s001.docx]

**Supplementary materials**

**Improved contraceptive use among women and men in Uganda between 1995-2016: a repeated cross-sectional population study**

**Table A. Any contraceptive use and demographic characteristics of women in Uganda across the study period (1995-2016).**

|  |  | **1995** | | | **2000/1** | | | **2006** | | | **2011** | | | **2016** | | |
| --- | --- | --- | --- | --- | --- | --- | --- | --- | --- | --- | --- | --- | --- | --- | --- | --- |
|  |  | **N** | **n** | **(%_w_)** | **N** | **n** | **(%_w_)** | **N** | **n** | **(%_w_)** | **N** | **n** | **(%_w_)** | **N** | **n** | **(%_w_)** |
| *Age (years)* | |  |  |  |  |  |  |  |  |  |  |  |  |  |  |  |
|  | 15-19 | 1,624 | 134 | (7.2) | 1,687 | 194 | (10.4) | 1,948 | 124 | (6.5) | 2,026 | 134 | (6.8) | 4,276 | 405 | (10.0) |
|  | 20-24 | 1,567 | 243 | (13.0) | 1,542 | 397 | (22.3) | 1,662 | 347 | (21.3) | 1,666 | 376 | (22.1) | 3,782 | 1,155 | (30.9) |
|  | 25-29 | 1,323 | 249 | (14.3) | 1,326 | 363 | (24.0) | 1,410 | 330 | (24.3) | 1,618 | 488 | (31.6) | 3,014 | 1,178 | (41.2) |
|  | 30-34 | 987 | 224 | (19.6) | 955 | 272 | (25.1) | 1,228 | 331 | (27.2) | 1,101 | 352 | (33.5) | 2,600 | 1,015 | (40.5) |
|  | 35-39 | 743 | 159 | (17.2) | 783 | 213 | (24.5) | 959 | 217 | (23.7) | 992 | 321 | (34.5) | 2,029 | 780 | (39.8) |
|  | ≥40 | 826 | 133 | (13.7) | 953 | 206 | (18.9) | 1,324 | 276 | (21.5) | 1,271 | 313 | (25.6) | 2,805 | 885 | (32.2) |
| *Highest educational attainment* | | | | |  |  |  |  |  |  |  |  |  |  |  |  |
|  | None | 1,808 | 136 | (7.3) | 1,459 | 179 | (11.7) | 1,768 | 173 | (11.6) | 1,332 | 160 | (16.6) | 2,071 | 426 | (23.4) |
|  | Primary | 3,901 | 575 | (13.0) | 4,098 | 810 | (18.0) | 4,922 | 868 | (18.0) | 4,820 | 1,042 | (21.9) | 10,893 | 3,127 | (29.0) |
|  | Higher^†^ | 1,361 | 431 | (29.0) | 1,688 | 655 | (36.8) | 1,841 | 564 | (31.1) | 2,522 | 782 | (30.6) | 5,542 | 1,865 | (34.5) |
| *Place of residence* | | |  |  |  |  |  |  |  |  |  |  |  |  |  |  |
|  | Urban | 2,439 | 627 | (26.9) | 2,416 | 838 | (36.1) | 1,450 | 478 | (32.5) | 2,562 | 810 | (33.0) | 4,379 | 1,445 | (34.1) |
|  | Rural | 4,631 | 515 | (11.0) | 4,830 | 807 | (16.9) | 7,081 | 1,127 | (16.9) | 6,112 | 1,174 | (21.3) | 14,127 | 3,973 | (28.9) |
| *Region* | |  |  |  |  |  |  |  |  |  |  |  |  |  |  |  |
|  | Central | 2,218 | 509 | (21.1) | 2,445 | 797 | (31.2) | 2,429 | 722 | (29.3) | 2,636 | 788 | (29.9) | 4,325 | 1,488 | (35.1) |
|  | East | 1,911 | 262 | (11.2) | 1,767 | 322 | (14.4) | 1,825 | 324 | (17.8) | 1,818 | 432 | (23.1) | 5,039 | 1,489 | (29.7) |
|  | North | 1,136 | 155 | (11.8) | 1,041 | 205 | (16.8) | 2,390 | 207 | (9.7) | 2,392 | 317 | (14.1) | 4,368 | 921 | (21.8) |
|  | West | 1,805 | 216 | (8.8) | 1,993 | 321 | (14.0) | 1,887 | 352 | (18.8) | 1,828 | 447 | (23.1) | 4,774 | 1,520 | (31.6) |

Note: ^†^Indicates secondary or higher educational attainment.

**Table B. Modern contraceptive use and demographic characteristics of women in Uganda across the study period (1995-2016).**

|  |  | **1995** | | | **2000/1** | | | **2006** | | | **2011** | | | **2016** | | |
| --- | --- | --- | --- | --- | --- | --- | --- | --- | --- | --- | --- | --- | --- | --- | --- | --- |
|  |  | **N** | **n** | **(%_w_)** | **N** | **n** | **(%_w_)** | **N** | **n** | **(%_w_)** | **N** | **n** | **(%_w_)** | **N** | **n** | **(%_w_)** |
| *Age (years)* | |  |  |  |  |  |  |  |  |  |  |  |  |  |  |  |
|  | 15-19 | 1,624 | 72 | (3.4) | 1,687 | 171 | (9.0) | 1,948 | 97 | (5.2) | 2,026 | 126 | (6.1) | 4,276 | 375 | (9.4) |
|  | 20-24 | 1,567 | 141 | (6.3) | 1,542 | 337 | (18.7) | 1,662 | 281 | (17.2) | 1,666 | 331 | (19.8) | 3,782 | 1,058 | (28.3) |
|  | 25-29 | 1,323 | 180 | (9.0) | 1,326 | 316 | (20.2) | 1,410 | 271 | (19.7) | 1,618 | 428 | (27.6) | 3,014 | 1,083 | (37.4) |
|  | 30-34 | 987 | 148 | (11.5) | 955 | 218 | (19.5) | 1,228 | 252 | (22.0) | 1,101 | 314 | (30.0) | 2,600 | 924 | (36.7) |
|  | 35-39 | 743 | 104 | (10.4) | 783 | 184 | (20.8) | 959 | 172 | (18.7) | 992 | 279 | (30.3) | 2,029 | 708 | (36.0) |
|  | ≥40 | 826 | 81 | (7.4) | 953 | 159 | (13.8) | 1,324 | 195 | (15.4) | 1,271 | 252 | (21.0) | 2,805 | 766 | (27.4) |
| *Highest educational attainment* | | | | |  |  |  |  |  |  |  |  |  |  |  |  |
|  | None | 1,808 | 53 | (2.4) | 1,459 | 135 | (8.6) | 1,768 | 117 | (8.2) | 1,332 | 141 | (14.6) | 2,071 | 378 | (20.6) |
|  | Primary | 3,901 | 358 | (7.1) | 4,098 | 668 | (14.6) | 4,922 | 688 | (14.3) | 4,820 | 926 | (19.5) | 10,893 | 2,871 | (26.6) |
|  | Higher^†^ | 1,361 | 315 | (20.2) | 1,688 | 581 | (32.1) | 1,841 | 463 | (25.2) | 2,522 | 663 | (26.1) | 5,542 | 1,665 | (30.5) |
| *Place of residence* | | |  |  |  |  |  |  |  |  |  |  |  |  |  |  |
|  | Urban | 2,439 | 493 | (22.1) | 2,416 | 754 | (32.9) | 1,450 | 414 | (28.2) | 2,562 | 695 | (28.5) | 4,379 | 1,297 | (30.5) |
|  | Rural | 4,631 | 233 | (4.8) | 4,830 | 631 | (13.2) | 7,081 | 854 | (12.9) | 6,112 | 1,035 | (18.8) | 14,127 | 3,167 | (26.1) |
| *Region* | |  |  |  |  |  |  |  |  |  |  |  |  |  |  |  |
|  | Central | 2,218 | 370 | (14.6) | 2,445 | 704 | (27.3) | 2,429 | 601 | (24.3) | 2,636 | 671 | (25.8) | 4,325 | 1,332 | (31.2) |
|  | East | 1,911 | 151 | (5.3) | 1,767 | 265 | (11.2) | 1,825 | 256 | (14.3) | 1,818 | 385 | (20.6) | 5,039 | 1,355 | (27.1) |
|  | North | 1,136 | 45 | (2.2) | 1,041 | 155 | (12.7) | 2,390 | 159 | (7.5) | 2,392 | 302 | (13.7) | 4,368 | 869 | (20.5) |
|  | West | 1,805 | 160 | (5.8) | 1,993 | 261 | (10.6) | 1,887 | 252 | (13.5) | 1,828 | 372 | (19.4) | 4,774 | 1,358 | (28.1) |

Note: ^†^Indicates secondary or higher educational attainment.

**Table C. Any contraceptive use and demographic characteristics of men in Uganda across the study period (1995-2016).**

|  |  | **1995** | | | **2000/1** | | | **2006** | | | **2011** | | | **2016** | | |
| --- | --- | --- | --- | --- | --- | --- | --- | --- | --- | --- | --- | --- | --- | --- | --- | --- |
|  |  | **N** | **n** | **(%_w_)** | **N** | **n** | **(%_w_)** | **N** | **n** | **(%_w_)** | **N** | **n** | **(%_w_)** | **N** | **n** | **(%_w_)** |
| *Age (years)* | |  |  |  |  |  |  |  |  |  |  |  |  |  |  |  |
|  | 15-19 | 375 | 31 | (7.8) | 437 | 60 | (13.2) | 582 | 67 | (10.7) | 562 | 82 | (13.0) | 1,270 | 272 | (22.9) |
|  | 20-24 | 379 | 95 | (24.6) | 337 | 113 | (30.8) | 397 | 116 | (29.6) | 340 | 128 | (38.1) | 944 | 451 | (49.1) |
|  | 25-29 | 381 | 91 | (19.7) | 310 | 98 | (31.4) | 351 | 96 | (27.7) | 365 | 128 | (36.0) | 740 | 366 | (47.7) |
|  | 30-34 | 256 | 79 | (28.8) | 274 | 89 | (25.8) | 358 | 106 | (28.5) | 310 | 95 | (30.5) | 737 | 332 | (44.7) |
|  | 35-39 | 249 | 83 | (27.0) | 223 | 59 | (24.8) | 318 | 81 | (24.2) | 284 | 93 | (32.9) | 497 | 212 | (42.6) |
|  | ≥40 | 356 | 83 | (18.9) | 356 | 92 | (23.0) | 497 | 136 | (29.7) | 434 | 141 | (33.3) | 1,148 | 469 | (42.2) |
| *Highest educational attainment* | | | | |  |  |  |  |  |  |  |  |  |  |  |  |
|  | None | 199 | 15 | (7.8) | 117 | 13 | (12.0) | 150 | 20 | (16.8) | 126 | 17 | (18.1) | 231 | 40 | (19.2) |
|  | Primary | 1,174 | 221 | (17.6) | 1,187 | 245 | (19.2) | 1,604 | 323 | (19.6) | 1,310 | 324 | (25.4) | 3,047 | 1,002 | (33.2) |
|  | Higher^†^ | 623 | 226 | (32.7) | 633 | 253 | (37.5) | 749 | 259 | (35.1) | 859 | 326 | (36.8) | 2,058 | 1,060 | (51.1) |
| *Place of residence* | | |  |  |  |  |  |  |  |  |  |  |  |  |  |  |
|  | Urban | 657 | 229 | (35.7) | 590 | 249 | (45.6) | 391 | 157 | (38.4) | 631 | 260 | (41.5) | 1,150 | 555 | (48.2) |
|  | Rural | 1,339 | 233 | (17.7) | 1,347 | 262 | (19.8) | 2,112 | 445 | (21.2) | 1,664 | 407 | (26.0) | 4,186 | 1,547 | (37.2) |
| *Region* | |  |  |  |  |  |  |  |  |  |  |  |  |  |  |  |
|  | Central | 641 | 178 | (22.7) | 673 | 255 | (36.7) | 724 | 251 | (34.0) | 672 | 261 | (37.0) | 1,258 | 579 | (37.0) |
|  | East | 546 | 114 | (20.1) | 458 | 97 | (17.2) | 536 | 140 | (25.6) | 499 | 145 | (28.3) | 1,450 | 526 | (28.3) |
|  | North | 331 | 85 | (23.8) | 269 | 48 | (14.5) | 661 | 110 | (17.0) | 601 | 136 | (25.8) | 1,249 | 439 | (25.8) |
|  | West | 478 | 85 | (14.8) | 537 | 111 | (19.2) | 582 | 101 | (17.2) | 523 | 125 | (22.8) | 1,379 | 558 | (22.8) |

Note: ^†^Indicates secondary or higher educational attainment.

**Table D. Modern contraceptive use and demographic characteristics of men in Uganda across the study period (1995-2016).**

|  |  | **1995** | | | **2000/1** | | | **2006** | | | **2011** | | | **2016** | | |
| --- | --- | --- | --- | --- | --- | --- | --- | --- | --- | --- | --- | --- | --- | --- | --- | --- |
|  |  | **N** | **n** | **(%_w_)** | **N** | **n** | **(%_w_)** | **N** | **n** | **(%_w_)** | **N** | **n** | **(%_w_)** | **N** | **n** | **(%_w_)** |
| *Age (years)* | |  |  |  |  |  |  |  |  |  |  |  |  |  |  |  |
|  | 15-19 | 375 | 22 | (5.0) | 437 | 55 | (12.2) | 582 | 65 | (10.4) | 562 | 81 | (12.8) | 1,270 | 255 | (21.6) |
|  | 20-24 | 379 | 66 | (15.0) | 337 | 98 | (26.6) | 397 | 104 | (26.5) | 340 | 114 | (33.7) | 944 | 419 | (45.4) |
|  | 25-29 | 381 | 54 | (9.7) | 310 | 74 | (23.9) | 351 | 84 | (24.8) | 365 | 110 | (32.2) | 740 | 333 | (43.2) |
|  | 30-34 | 256 | 39 | (10.8) | 274 | 66 | (17.7) | 358 | 92 | (25.7) | 310 | 82 | (25.6) | 737 | 285 | (37.1) |
|  | 35-39 | 249 | 48 | (13.6) | 223 | 42 | (17.5) | 318 | 60 | (19.0) | 284 | 76 | (27.5) | 497 | 186 | (38.0) |
|  | ≥40 | 356 | 44 | (9.8) | 356 | 68 | (16.1) | 497 | 101 | (22.1) | 434 | 115 | (27.1) | 1,148 | 419 | (37.6) |
| *Highest educational attainment* | | | | |  |  |  |  |  |  |  |  |  |  |  |  |
|  | None | 199 | 5 | (2.3) | 117 | 8 | (6.9) | 150 | 12 | (11.0) | 126 | 14 | (14.9) | 231 | 36 | (18.2) |
|  | Primary | 1,174 | 101 | (6.8) | 1,187 | 178 | (13.6) | 1,604 | 266 | (16.6) | 1,310 | 281 | (22.0) | 3,047 | 907 | (30.0) |
|  | Higher^†^ | 623 | 167 | (23.1) | 633 | 217 | (32.4) | 749 | 228 | (30.9) | 859 | 283 | (32.0) | 2,058 | 954 | (45.7) |
| *Place of residence* | | |  |  |  |  |  |  |  |  |  |  |  |  |  |  |
|  | Urban | 657 | 167 | (27.8) | 590 | 220 | (41.9) | 391 | 148 | (36.2) | 631 | 225 | (36.2) | 1,150 | 509 | (44.0) |
|  | Rural | 1,339 | 106 | (7.6) | 1,347 | 183 | (14.0) | 2,112 | 358 | (17.5) | 1,664 | 353 | (22.5) | 4,186 | 1,388 | (33.2) |
| *Region* | |  |  |  |  |  |  |  |  |  |  |  |  |  |  |  |
|  | Central | 641 | 140 | (18.0) | 673 | 225 | (32.8) | 724 | 230 | (31.4) | 672 | 231 | (33.2) | 1,258 | 538 | (41.5) |
|  | East | 546 | 66 | (9.7) | 458 | 81 | (14.4) | 536 | 105 | (19.5) | 499 | 119 | (22.9) | 1,450 | 468 | (31.7) |
|  | North | 331 | 15 | (3.7) | 269 | 27 | (6.7) | 661 | 83 | (13.2) | 601 | 122 | (23.0) | 1,249 | 409 | (33.3) |
|  | West | 478 | 52 | (8.2) | 537 | 70 | (10.3) | 582 | 88 | (14.9) | 523 | 106 | (19.5) | 1,379 | 482 | (35.2) |

Note: ^†^Indicates secondary or higher educational attainment.
